# Dual‐Graded Microstructure Engineering for Flexible Piezoresistive Sensors with High Sensitivity and Broad Linear Range in Physiological Monitoring

**DOI:** 10.1002/advs.202507135

**Published:** 2025-07-14

**Authors:** Ningning Bai, Dandan Xu, Zhuang Su, Gangqiang Li, Lilong He, Yanli Chen, Chengxi Guo, Linxuan Zhou, Xianming Qin, Ji Zhang, Daowei Wu, Weidong Wang

**Affiliations:** ^1^ School of Mechano‐Electronic Engineering Xidian University Xi'an 710071 P. R. China; ^2^ State Key Laboratory of Electromechanical Integrated Manufacturing of High‐Performance Electronic Equipment Xi'an 710071 P. R. China; ^3^ Xi'an Chuanglian Ultrasound Technology Co. Ltd Xi'an 710065 P. R. China; ^4^ Xi'an Microelectronics Technology Institute Xi'an 710054 P. R. China

**Keywords:** dual‐graded microstructures, flexible piezoresistive sensor, high‐sensitive and linear response, physiological monitoring, TPU@MWCNTs sensitive films

## Abstract

Flexible piezoresistive sensors that offer both high sensitivity and a broad linear detection range are highly desirable for wearable health monitoring, as they facilitate simplified circuit design and enable accurate detection of subtle physiological signals. However, existing sensors typically encounter an intrinsic trade‐off between sensitivity and linearity, primarily due to structural stiffening under increasing pressure. Here, a flexible piezoresistive pressure sensor featuring dual‐graded microstructures (DGM) is reported, formed by embedding multi‐walled carbon nanotubes (MWCNTs) into a thermoplastic polyurethane matrix. Leveraging the synergistic effects of progressive structural deformation and MWCNTs‐induced tunneling conduction, the sensor achieves a high sensitivity of 69.8 kPa⁻¹ and a broad linear sensing range up to 300 kPa (*R* ^2^ ≈ 0.998). The sensor also exhibits rapid response‐relaxation time (totaling 5 ms), stable high‐frequency detection up to 200 Hz, and good stability over 5 000 repeated loading cycles. Demonstrations in physiological monitoring confirm the sensor's capability to precisely capture detailed radial pulse waveforms, respiratory rhythms, and subtle heartbeat‐induced vibrations. Both a scalable, cost‐effective structural fabrication and good overall sensing performance establish the DGM‐based sensor as a promising candidate for advanced wearable healthcare monitoring devices.

## Graphical abstract

A flexible piezoresistive pressure sensor with dual‐graded microstructures achieves both high sensitivity (69.8 kPa⁻¹) and a broad linear range up to 300 kPa (*R* ^2^ = 0.997). Synergizing structural deformation and tunneling conduction, it enables rapid, stable, and accurate detection of subtle physiological signals, offering a scalable and cost‐effective solution for next‐generation wearable health monitoring

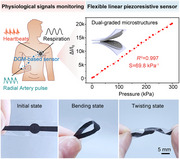

## 1 Introduction

Flexible pressure sensors, which convert mechanical deformation into electrical signals,^[^ ^1^, ^2^, ^3^, ^4^ ^]^ have become essential components in wearable electronics and personalized healthcare due to their ability to conform precisely to complex geometries and dynamically monitor physiological parameters in real‐time.^[^ ^5^, ^6^, ^7^ ^]^ By conformally integrating with curved biological surfaces, these sensors enable precise and continuous monitoring of vital signs, including pulse, respiration, and heartbeats.^[^ ^8^, ^9^ ^]^ The rapid advancement of wearable devices and personalized healthcare has further heightened the demand for flexible pressure sensors possessing both high sensitivity and a wide linear detection range.^[^ ^10^, ^11^ ^]^ High sensitivity ensures precise detection of subtle physiological changes, while a broad linear response allows sensor outputs to remain directly proportional to the applied pressures, thereby simplifying signal analysis without the need for complex data processing.^[^ ^12^ ^]^

Flexible pressure sensors can be classified into capacitive, piezoelectric, triboelectric, and piezoresistive types according to their sensing mechanisms.^[^ ^13^ ^]^ Among these, piezoresistive sensors have received extensive attention owing to their relatively simple device structures, low‐cost fabrication processes, straightforward signal readout, and capability for miniaturization.^[^ ^14^ ^]^ Typically, piezoresistive pressure sensors operate by converting mechanical stimuli into measurable changes in resistance, driven by variations in internal conductive pathways or structural deformation.^[^ ^15^ ^]^ Extensive efforts have beendirected toward structural engineering and material innovation to enhance piezoresistive sensors performance. Various conductive fillers, such as graphene,^[^ ^16^, ^17^, ^18^ ^]^ carbon nanotubes (CNTs),^[^ ^3^, ^19^ ^]^ MXenes,^[^ ^20^, ^21^, ^22^ ^]^ and metal nanoparticles,^[^ ^23^, ^24^ ^]^ have been incorporated into elastomeric matrices as internal conductive pathways, thereby effectively enhancing both the mechanical robustness and electrical conductivity of the sensors. Concurrently, engineering various microstructures,^[^ ^25^, ^26^, ^27^, ^28^, ^29^, ^30^, ^31^, ^32^ ^]^ including micropyramids,^[^ ^25^, ^26^ ^]^ hierarchical structures,^[^ ^27^, ^28^ ^]^ porous networks,^[^ ^29^, ^30^ ^]^ and bionic microstructures,^[^ ^31^, ^32^ ^]^ has been employed to promote structural deformation under applied pressure, thereby increasing the sensor's sensitivity and extending sensing range. Nevertheless, these approaches frequently encounter a fundamental trade‐off between high sensitivity and a broad linear sensing range.

Recently, hybrid microstructures have been engineered to mitigate structural stiffening and improve compressibility, aiming to effectively balance sensitivity and linear sensing range in flexible piezoresistive pressure sensors.^[^ ^33^, ^34^, ^35^, ^36^, ^37^, ^38^, ^39^, ^40^, ^41^, ^42^, ^43^, ^44^ ^]^ Pyramidal carbon foam arrays with tunable stiffness, designed by theoretical exponential decay resistance models, achieved a sensitivity of 24.6 kPa⁻¹ within an ultra‐wide linear range (≤1.4 MPa).^[^ ^33^ ^]^ Inspired by biological systems, anisotropic conductive foams constructed by directional freezing and ultrasonic‐assisted dip‐coating methods demonstrate a broad linear sensing range at pressures up to 500 kPa, coupled with low detection limits, highlighting the advantages of hierarchical conductive networks.^[^ ^34^ ^]^ Dual‐conductive‐layer strategies incorporating porous and interlocking structures have demonstrated high sensitivity (924.4 kPa⁻¹) though only within a limited pressure range of up to 70 kPa.^[^ ^35^ ^]^ Additionally, dual‐layer Janus architectures with graded electrical conductivities offer segmented conductive pathways, achieving a wide linear sensing range (up to 3.8 MPa) while maintaining a moderate sensitivity of ≈4 kPa⁻¹.^[^ ^36^ ^]^ Although these strategies have improved sensor sensitivity or expanded the linear sensing range to some extent, inherent limitations still persist: high sensitivity typically remains confined to low or moderate pressures, whereas broader linear ranges usually compromise sensitivity. Additionally, multilayered or porous structures increase device thickness, adversely affecting flexibility and portability. Thus, developing new strategies to simultaneously achieve high sensitivity and a broad linear sensing range without sacrificing device flexibility and ease of fabrication remains highly desirable.

In this study, we reported a piezoresistive flexible sensor based on dual‐graded microstructures (DGM) fabricated by incorporating conductive multi‐walled carbon nanotubes (MWCNTs) into thermoplastic polyurethane (TPU), which achieves high sensitivity (69.8 kPa⁻¹) and high linearity (linear correlation coefficient *R* ^2^≈0.998) over a broad linear sensing range up to 300 kPa. The high linearity comes from the synergistic effects of significant changes in contact area based on DGM inducing variations in contact resistance (*R*_c_) and film resistance (*R*_f_) resulting from the conductive filler MWCNTs. Additionally, the DGM configuration enables an ultrafast total response‐relaxation time of 5 ms, and thus the sensor can detect vibration frequencies up to 200 Hz. The DGM‐based sensor also exhibited good work stability during prolonged dynamic monitoring under cyclic loading/unloading of 300 kPa (≈5 000 cycles). Its good comprehensive sensing performance allows the sensor to precisely capture subtle physiological signals, such as pulse, respiration, and heartbeats, positioning it as a promising solution for real‐time health monitoring applications.

## 2 Results and Discussion

### 2.1 Design and Preparation of the DGM‐Based Flexible Piezoresistive Pressure Sensor

**Figure 1:**
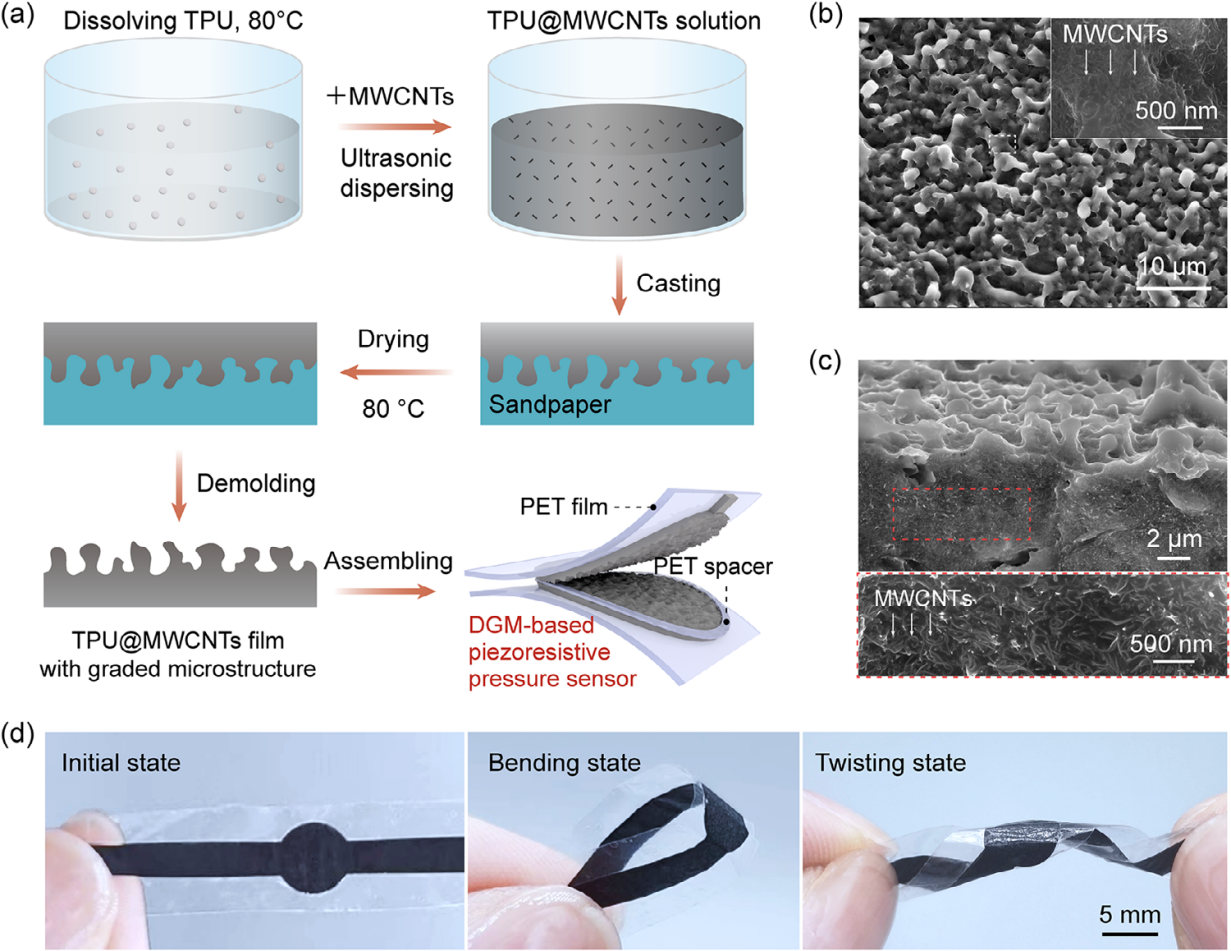
Design and preparation of the DGM‐based flexible piezoresistive pressure sensor. a) Schematic of the sensor fabrication process. b) Top‐view and c) cross‐sectional scanning electron microscopy (SEM) images of the TPU@MWCNTs sensitive film, with insets showing magnified views of the respective regions. d) Photographs of the pressure sensor in initial, bending, and twisting states, respectively.

In this study, the sensor was fabricated using a simple template‐assisted method. The elastic TPU was chosen to serve as the flexible polymer matrix, while highly conductive MWCNTs act as the conductive fillers. As illustrated in **Figure** **1**a, TPU was first dissolved in solvent to form a homogeneous solution, and MWCNTs were then added and uniformly dispersed via ultrasonication. The obtained TPU@MWCNTs solution was cast onto sandpaper with a microstructured surface (Figure S3, Supporting Information) and subsequently cured to form a sensitive film with graded microstructures. After demolding, two sensitive films were assembled face‐to‐face with their microstructured sides in contact, separated by an ultrathin polyethylene terephthalate (PET) spacer ring. The assembly was then securely sealed using transparent PET adhesive tape to form the DGM‐based flexible piezoresistive pressure sensor, hereafter referred to as the DGM‐based sensor. As shown in Figure 1b,c, the TPU@MWCNTs film features a graded microstructure composed of microscale ridges and grooves, with MWCNTs uniformly dispersed within the TPU matrix (Figure S4, Supporting Information). Such a graded microstructure combined with the uniform distribution of conductive fillers enhance interfacial compressibility and ensure a stable conductive network, which are essential for achieving both high sensitivity and a broad linear sensing range.

Notably, the DGM‐based sensor exhibits exceptional flexibility and mechanical robustness, allowing for bending and even twisting without delamination (Figure 1d). Compared to the fabrication methods of most conventional flexible linear pressure sensors, the approach proposed in this study is cost‐effective, scalable, and easy to implement, making it highly suitable for applications in wearable electronics and intelligent sensing.

### 2.2 Design Principle and Sensing Mechanism of DGM‐based Flexible Piezoresistive Pressure Sensor

**Figure 2:**
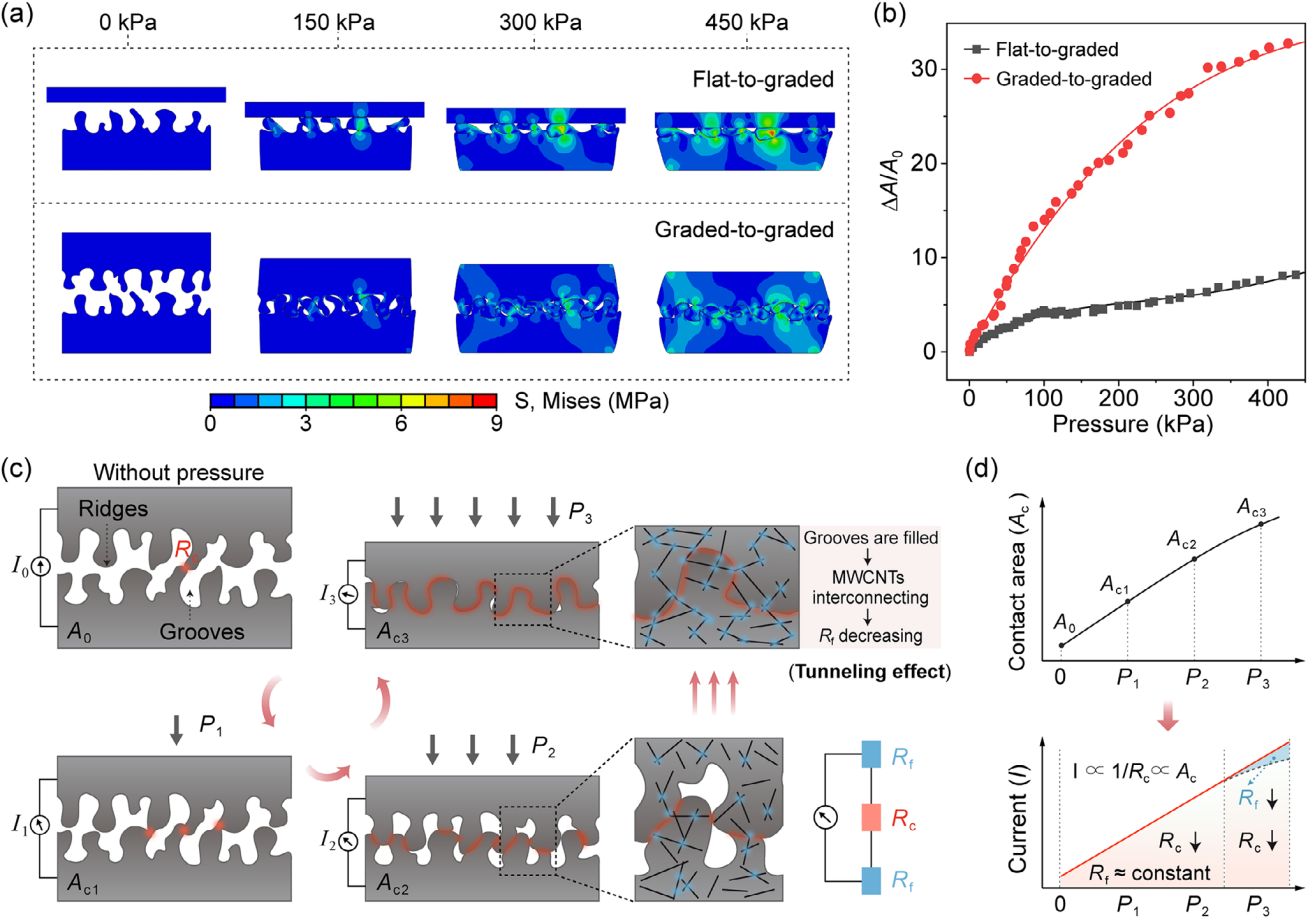
Design principle and sensing mechanism of the DGM‐based flexible piezoresistive sensor. a) Stress distribution of simulation of flat‐to‐graded and graded‐to‐graded interfacial configurations under pressures up to 450 kPa. b) Normalized change in contact area for the two configurations under a broad sensing range over 450 kPa. c) Schematic of the sensing mechanism of the sensor. d) Schematic of contact area and resistance variation with increasing pressure.

Most flexible pressure sensors exhibit nonlinear responses over a wide pressure range due to the structural stiffening effect of soft materials, which leads to a sharp decline in compressibility as the pressure increases.^[^ ^4^, ^45^, ^46^ ^]^ To mitigate the limitation, finite element analysis (FEA) simulations were performed to investigate two interface configurations featuring distinct compressibility characteristics and microstructure dimensions (**Figure** **2**a): a flat‐to‐graded configuration, where a flat film contacts a graded microstructured film; and a graded‐to‐graded configuration (termed DGM), comprising two graded microstructured films aligned face‐to‐face.

In the flat‐to‐graded configuration, micro ridges with different heights progressively contact the flat film under low pressures, causing a gradual increase in contact area (*A*_c_). However, as the pressure exceeds ≈100 kPa, the micro ridges begin to compress extensively and eventually become flattened into the grooves, leading to pronounced stress concentration and structural stiffening effects. Consequently, the rate at which the *A*_c_ increases significantly diminishes at higher pressures, resulting in an overall nonlinear response (Figure 2b, Table S1, Supporting Information). In contrast, the graded‐to‐graded configuration exhibits greater effective compressibility, as the micro‐ridges on opposing graded surfaces progressively interlock and fill the corresponding grooves throughout a broad pressure range. This continuous structural deformation maintains high interfacial compressibility and enables a stable, near‐linear increase in *A*_c_ over a broad pressure range.

To further elucidate the linear sensing mechanism of the DGM‐based sensor, a schematic analysis illustrates the interplay between structural deformation and tunneling effects of conductive fillers (Figure 2c). According to Ohm's law and the definition of resistance, the sensor's output current (*I*) under a constant voltage (*V*) is proportional to *A*_c_ and inversely proportional to the film thickness (*L*), expressed as $I=\frac{V}{R}=\frac{V\cdot A_{c}}{\rho \cdot L}$, where *ρ* is resistivity.

In the unloaded state, only a limited number of the tallest micro ridges on the DGM interface come into contact, yielding a minimal initial contact area (*A*_0_), which leads to a high *R*_c_ and low initial current (*I*_0_). Upon applying low pressure, more micro ridges engage, gradually increasing *A*_c_ (Figure 2d). In the medium pressure range, the DGM adaptively deforms and forms interlocking contacts, with minimal variation in film thickness, leading to an approximately linear increase in *A*_c_ with pressure. At high pressures, the grooves between micro ridges become progressively filled through interlocking with opposing ridges, causing the interface to exhibit solid‐like compressive behavior, which limits further deformation and slows the growth of *A*_c_. Meanwhile, the reduced film thickness facilitates interconnections between adjacent MWCNTs and enhances the tunneling effect, thereby lowering the film resistance *R_f_*. This compensates for the slower increase in *A*_c_, maintaining an approximately linear current response across the full pressure range.

As a result, the proposed design is expected to enable the DGM‐based sensor with high sensitivity and linearity across a broad range, through the synergistic interplay between the DGM configuration and pressure‐induced tunneling conduction.

### 2.3 Sensing Properties of the DGM‐Based Flexible Piezoresistive Pressure Sensor

**Figure 3:**
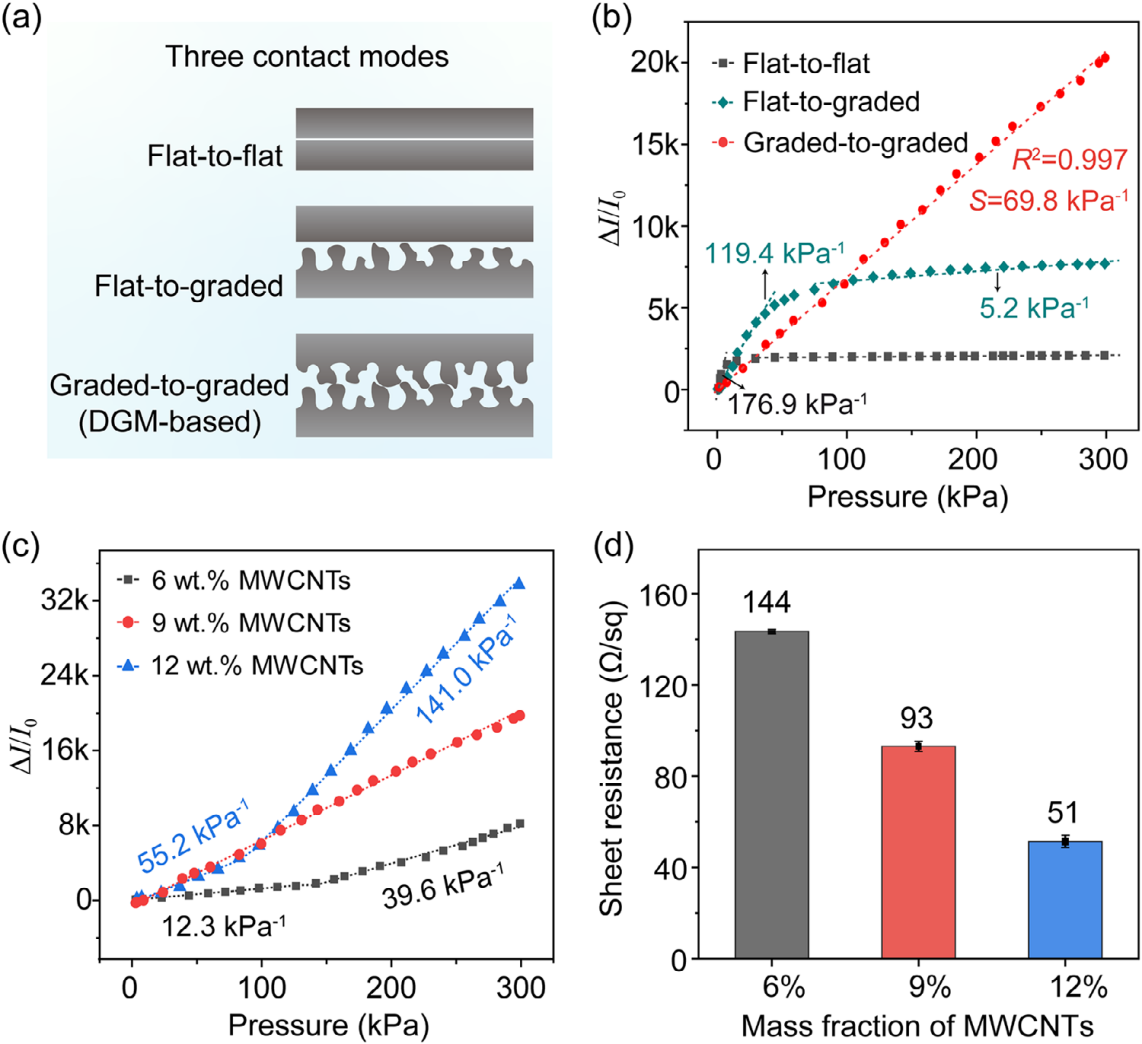
Sensor sensitivity and resistance of TPU@MWCNTs sensitive films. a) Three contact modes of the sensing interface: flat‐to‐flat, flat‐to‐graded, and graded‐to‐graded. b) Change in current for the sensors (9 wt.% MWCNTs) with three different contact modes under pressures up to 300 kPa. c) Change in current for the DGM‐based sensors with the TPU@MWCNTs sensitive films containing MWCNTs at mass fractions of 6 wt.%, 9 wt.%, and 12 wt.% under pressures up to 300 kPa. d) Sheet resistance of TPU@MWCNTs sensitive films with varying mass fractions of MWCNTs.

To validate the effectiveness of the proposed design, the sensing performance of the DGM‐based sensor was systematically characterized in terms of sensitivity, linearity, and sensing range. Sensitivity (*S*) was defined as *S* = δ(Δ*I*/*I*_0_)/δ*P*, where Δ*I* is the change in current under an applied pressure *P*, and *I*_0_ is the initial current. To verify the enhanced sensing performance of the DGM configuration, three distinct interfacial contact modes were investigated (**Figure** **3**a): flat‐to‐flat (two flat films in contact), flat‐to‐graded (as illustrated in Figure 2a), and the DGM. As shown in Figure 3b, the sensor with a flat‐to‐flat configuration exhibited high sensitivity at low pressures (179.6 kPa⁻¹ below 10 kPa) due to the large *A*_0_; however, the response quickly saturated at higher pressures, leading to highly nonlinear behavior. The flat‐to‐graded configuration demonstrated improved sensitivity retention across a broader pressure range (5.2 kPa⁻¹ within 40–300 kPa) through stepwise microstructure engagement, yet still exhibited a nonlinear response trend. In contrast, the DGM‐based sensor maintained both high sensitivity (69.8 kPa⁻¹) and linearity (*R* ^2^ = 0.998) over a wide pressure range up to 300 kPa, consistent with the design rationale of the DGM concept.

The DGM‐based sensors constructed with different microstructure sizes were also studied to assess the impact on sensing performance (Figure S5, Supporting Information). The results demonstrate that sensors with finer graded microstructures exhibit more compact and continuous interfacial interlocking under pressure, which enhances structural compressibility (Figure S6a, Supporting Information). This leads to a more stable increase in contact area during loading (Figure S6b, Table S1, Supporting Information), ultimately contributing to higher sensitivity and an extended linear response range compared to those with a larger‐scale graded microstructured configuration (Figure S6c, Supporting Information).

For piezoresistive sensors, the content of conductive fillers influences the mechano‐electronic behavior of the sensing material. In this study, we systematically investigated the effect of MWCNTs loading on sensor performance. DGM‐based sensors were fabricated using sensing films with MWCNTs mass fractions of 6 wt.%, 9 wt.%, and 12 wt.%, and their sensing performance was evaluated over a pressure range up to 300 kPa (Figure 3c).

The sensor loaded with 12 wt.% MWCNTs exhibited the highest sensitivity; however, its pressure response showed a distinct two‐stage behavior. The sensitivity was 55.2 kPa⁻¹ at pressures below 100 kPa, but markedly increased to 141 kPa⁻¹ in the high pressure range (100–300 kPa). This nonlinearity is primarily attributed to the low initial *R_f_* of the film at high filler content (Figure 3d), which enhances the tunneling effect between adjacent MWCNTs under high pressure, thereby increasing current output. Despite its superior sensitivity, mechanical characterization showed that the sensing film with 12 wt.% MWCNTs exhibited low elongation at break, indicating poor mechanical compliance (Figure S7, Supporting Information). The 6 wt.% MWCNTs‐loaded sensor exhibited the lowest sensitivity (12.3 kPa⁻¹) at low pressures due to insufficient conductive network formation and limited *R*_c_ modulation. At elevated pressures, the enhanced compressibility of the low‐modulus matrix facilitated MWCNT contact and tunneling conduction, decreasing *R_f_* and increasing sensitivity to 39.6 kPa⁻¹. Although the film showed greater flexibility, favorable for wearable applications, the low filler content ultimately limited its sensing performance.

Notably, the sensor with 9 wt.% MWCNTs exhibited well‐balanced mechano‐electronic performance, which can be attributed to the appropriate modulus of the sensing film that accommodates deformation of the DGM (affecting *R_c_*), while simultaneously enabling moderate tunneling effects to effectively modulate *R_f_*. The synergistic regulation achieved both high sensitivity and a broad linear response range. Moreover, the sensing film with 9 wt.% MWCNTs offered a favorable trade‐off between mechanical robustness and flexibility, supporting its practical application.

Overall, the MWCNT fillers content plays a noteworthy role in tuning the comprehensive performance of DGM‐based TPU@MWCNTs piezoresistive sensors. Insufficient filler content fails to establish an effective conductive network, thereby limiting sensitivity, whereas excessive loading enhances electrical conductivity but compromises mechanical compliance and linear response. A filler loading of 9 wt.% achieves an optimal trade‐off between *R_c_* and tunneling‐induced *R_f_*, yielding a synergistic enhancement in sensitivity, linear response, and mechanical flexibility. The findings provide a material‐level rationale for selecting filler content to achieve balanced mechano‐electronic performance in flexible pressure sensors.

### 2.4 Dynamic Response and Stability of the DGM‐Based Flexible Piezoresistive Pressure Sensor

**Figure 4:**
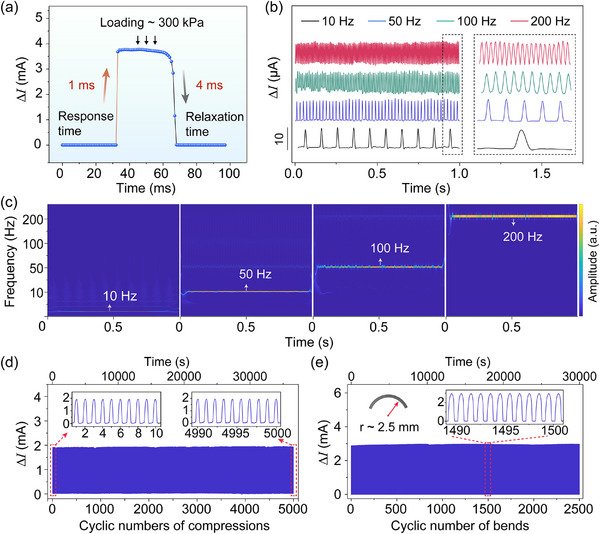
Dynamic response performance and stability of the DGM‐based pressure sensor. a) Response and release times at a loading of ≈300 kPa. b) Time dependent variation of current at different vibration frequencies. c) Wavelet transform of vibration signals shown in (b). d) Working stability test over 5 000 cycles under a high pressure of 300 kPa. e) Bending signal stability under 2 500 bending cycles at a radius (*r*) of ≈2.5 mm.

Dynamic response and mechanical stability are also key performance metrics for pressure sensors, especially in real‐time physiological monitoring. Embedding microstructures in the sensing layer enhances the sensor's response and relaxation speed by reducing interfacial adhesion and promoting rapid elastic energy storage and release.^[^ ^4^, ^45^, ^46^, ^47^ ^]^ Here, the response and relaxation times were measured by manually applying and releasing a compressive load using a 2 mm‐diameter flat metal rod. The rising and falling edges of the signal correspond to the response and relaxation times, respectively. Benefiting from the optimized graded‐to‐graded configuration, the piezoresistive sensor achieved a rapid response time of 1 ms and a relaxation time of 4 ms under an applied pressure of ≈300 kPa (**Figure** **4**a), enabling prompt signal acquisition and release. This rapid, dynamic behavior enables the sensor to detect high‐frequency mechanical stimuli. As illustrated in Figure 4b, the sensor accurately detected vibrations at 10, 50, 100, and up to 200 Hz without signal distortion or delay. The corresponding time–frequency map further confirms the sensor's ability to resolve transient vibration frequencies with high temporal resolution (Figure 4c).

Mechanical stability under repetitive loading is essential for evaluating the long‐term reliability of flexible electronics. The DGM‐based sensor was therefore subjected to over 5 000 compression–release cycles at a peak pressure of 300 kPa. As shown in Figure 4d, the sensor exhibited stable signal output throughout the test, with no noticeable drift or degradation, indicating good durability. The sensor also demonstrated dynamic mechanical stability under cyclic loading at 5 Hz (Figure S8, Supporting Information), which is important for dynamic applications such as physiological monitoring. In addition, bending tests conducted at a radius (*r*) of ≈2.5 mm for over 2 500 cycles demonstrated stable signal response and recovery (Figure 4e), further validating the sensor's mechanical robustness under flexural deformation. The negligible fluctuations in signal amplitude during both compression and bending cycles highlights the structural resilience and reliable performance of the DGM‐based sensor under complex loading conditions.

As summarized in Table S2 (Supporting Information), our DGM‐based sensor demonstrates high sensitivity, a broad linear operating range, and fast response‐relaxation time, outperforming most existing piezoresistive sensors reported in the literature.^[^ ^9^, ^33^, ^34^, ^35^, ^36^, ^37^, ^38^, ^39^, ^40^, ^41^, ^42^, ^43^, ^44^ ^]^ The linear sensing factor, *S*_P_, which is the product of sensitivity and linear sensing range (*S*_P_ = *S*⋅Δ*P*), indicates better linear sensing performance with higher values.^[^ ^46^ ^]^ While a few reported sensors have slightly higher *S*_P_ values than our sensor, their slower response‐relaxation times suggests that our sensor has better dynamic monitoring capabilities.^[^ ^33^ ^]^ With its good comprehensive performance and cost‐effective fabrication technique, the DGM‐based sensor offers competitive advantages in linear sensing and dynamic response compared to most reported flexible piezoresistive sensors.

### 2.5 Physiological Monitoring by the DGM‐Based Flexible Piezoresistive Pressure Sensor

The DGM‐based sensor demonstrates good comprehensive sensing performance, including high sensitivity, a broad linear response range, fast response speed, and good stability, enabling the detection of weak physiological signals. In this section, we systematically evaluated the sensor's performance in monitoring radial artery pulse, respiration, and heartbeats.

#### 2.5.1 Radial Pulse Monitoring

**Figure 5:**
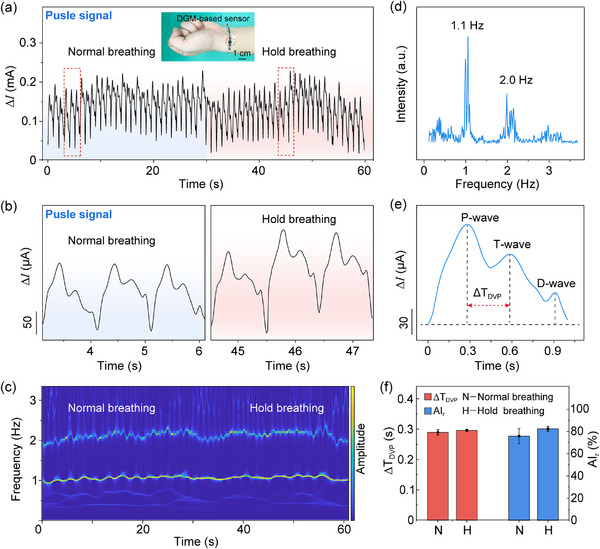
Arterial pulse monitoring using the DGM‐based flexible piezoresistive sensor. a) Radial artery signals during normal breathing and breath‐holding. b) Enlarged view of the pulse waveforms from panel (a). c) Wavelet transform of the pulse signals shown in panel (a). d) Pulse frequency during normal breathing and hold breathing. e) A single pulse wave showing the P‐, T‐, and D‐waves. f) Statistical results of ΔT_DVP_ and AI_r_ for the pulse at normal breathing and hold breathing.

As shown in **Figure** **5**a, radial artery pulse signals were recorded from an asymptomatic male subject during normal breath and breath holding (≈30 s). Compared to regular respiration, breath holding resulted in noticeably elevated pulse amplitudes (Figure 5b), potentially reflecting short‐term hemodynamic compensation, such as increased peripheral vascular resistance or transient blood pressure elevation in response to mild hypoxia. Wavelet‐based time–frequency analysis revealed that the dominant pulse frequency remained stable across both breathing states (Figure 5c), suggesting that the brief apnea period did not significantly alter heart rate. The observed consistency is likely due to the brief duration of breath holding and the body's autonomic regulation mechanisms, which maintain peripheral hemodynamics balance under mild physiological perturbations. Fourier spectral analysis identified two prominent frequency components at approximately 1.1 and 2.0 Hz, corresponding to the primary pulse frequency and its second harmonic, respectively (Figure 5d). Furthermore, following physical exertion, the normal breathing rate increased from 1.1 Hz (66 bpm) to 1.4 Hz (84 bpm), reflecting the body's increased oxygen demand post‐exercise (Figure S9, Supporting Information). The above time‐domain signal and frequency‐domain signal analysis demonstrated the sensor's capability to capture detailed frequency features of the pulse waveform, demonstrating its effectiveness in resolving subtle multi‐frequency pulse fluctuations.

Moreover, the sensor's high sensitivity enabled precise detection of distinct waveform parameters, including pulse amplitude, pulse rate, and vascular tension indices. Specifically, each pulse cycle exhibited three clearly distinguishable components—the percussion wave (P‐wave), tidal wave (T‐wave), and dicrotic wave (D‐wave)—which were well resolved in the magnified pulse signal acquired by the sensor (Figure 5e). The P‐wave arises from the rapid ejection of blood from the left ventricle during early systole; the T‐wave results from late‐systolic reflections in the upper limb arteries; and the D‐wave is associated with diastolic reflections, typically linked to aortic valve closure and wave return from the lower body.^[^ ^48^ ^]^To quantitatively assess subtle changes in vascular function, two key parameters were extracted from the pulse waveform: the radial artery augmentation index (AI_r_) and ΔT_DVP_. AI_r_ is defined as the amplitude ratio of the T‐wave to the P‐wave, while ΔT_DVP_ represents the temporal delay between the P‐wave and T‐wave peaks.^[^ ^49^ ^]^ During normal breathing, AI_r_ was 75.8%, and ΔT_DVP_ was 289 ms. Under breath‐holding conditions, AI_r_ increased to 82.4%, and ΔT_DVP was_ slightly prolonged to 296 ms (Figure 5f). These systematic variations suggest that transient hypoxia during breath holding enhances peripheral wave reflection and delays arterial response times, likely indicating transient increases in arterial stiffness or peripheral vasoconstriction under mild hypoxic stress.

#### 2.5.2 Dynamic Respiratory Monitoring

**Figure 6:**
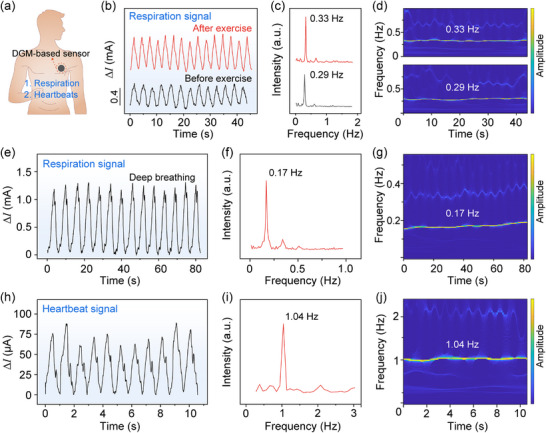
DGM‐based flexible piezoresistive pressure sensor for respiration and heartbeat monitoring. Schematic showing the placement of the DGM‐based sensor on the human body for respiration and heartbeat monitoring. b) Time‐dependent current signals of respiration before and after exercise. c) Extracted respiration frequencies corresponding to the signals in panel (b). d) Wavelet transform of the respiration signals shown in (b). e) Time‐dependent current signals during rapid breathing. f) Rapid breathing frequencies extracted from the signals in (e). g) Wavelet transform of the rapid respiration signals shown in panel (e). h) Time‐dependent current signals of heartbeat. i) Heartbeat frequency extracted from the signals in panel (h). j) Wavelet transform of the heartbeat signals shown in panel (h).

In addition to pulse signal detection, we further evaluated the sensor's capability in dynamic cardiopulmonary monitoring by attaching the device to a male subject's chest (**Figure** **6**a). Continuous monitoring was conducted before and after the subject performed 25 push‐ups. As shown in Figure 6b, both the amplitude and frequency of the respiratory signal increased following physical exertion, with the respiratory rate increasing from 0.29 to 0.33 Hz (17–20 bpm). The observed increase reflects deeper and more rapid breathing postexercise (Figure 6c), primarily resulting from elevated metabolic demands and increased carbon dioxide accumulation. Wavelet‐based time–frequency analysis further verified that the respiratory frequency remained consistently centered ≈0.29 and 0.33 Hz, respectively, during the ≈45 s monitoring windows before and after exercise (Figure 6d), confirming the sensor's ability to accurately track both long‐term respiratory trends and transient fluctuations in breathing dynamics with high temporal resolution.

To investigate broader respiratory dynamics, we instructed the subject to perform slow and deep inhalation–exhalation cycles under controlled conditions. As shown in Figure 6e, the sensor reliably detected these enhanced respiratory efforts, as evidenced by a substantial increase in signal amplitude. Notably, the corresponding Fourier spectrum (Figure 6f) revealed two distinct frequency components at ≈0.17 Hz (corresponding to ≈10 bpm) and 0.34 Hz. During normal respiration, the second sub‐peak is typically weaker and often obscured within the signal due to its low amplitude. In contrast, under deep and forceful breathing, both sub‐peaks become clearly distinguishable in the time domain, giving rise to dual frequency components in the corresponding Fourier spectrum. These two frequency components are attributed to the basic respiratory rhythm and its harmonic, respectively, which emerge under slow, forceful breathing due to the characteristics of respiratory airflow and thoracic motion. The wavelet‐based time–frequency analysis further delineates the temporal evolution of these components (Figure 6g), demonstrating that the sensor could discern detailed respiratory patterns with both high temporal and frequency resolution.

#### 2.5.3 Heartbeat Signal Detection

In addition to respiration, the sensor demonstrated the ability to detect fine chest vibrations associated with cardiac activity. The smaller displacement of the thoracic wall during heartbeats, compared to breathing, resulted in lower signal amplitudes. Nonetheless, the sensor successfully captured heartbeat‐induced mechanical oscillations under resting conditions (Figure 6h). Notably, each heartbeat cycle exhibited a tri‐phasic waveform, featuring one dominant peak and two smaller sub‐peaks. The primary peak likely reflects rapid ventricular ejection, while the secondary features may correspond to aortic valve closure and subsequent pressure reflections.^[^ ^50^ ^]^ The observation suggests that the sensor is capable of resolving complex biomechanical signatures associated with cardiac mechanical events, beyond simple beat‐to‐beat timing. Fourier analysis of the acquired signals revealed a dominant frequency peak at ≈1.04 Hz (≈62 bpm), which corresponds to periodic chest wall vibrations induced by cardiac activity (Figure 6i). This frequency falls within the normal resting heart rate range (60–100 bpm) and suggests that the sensor is capable of detecting heartbeat‐associated mechanical responses. The corresponding time–frequency analysis confirmed stable periodicity and temporal coherence of the heartbeat signals across the measurement window (Figure 6j), further demonstrating the effectiveness of the DGM‐based sensor in detecting weak biomechanical signals.

To further verify dynamic responsiveness, the sensor was applied to throat vibration monitoring, capturing distinct signals during swallowing and speech (e.g., pronouncing the word “world”), with frequency components up to 200 Hz (Figure S10, Supporting Information), demonstrating its suitability for tracking rapid physiological motions. Collectively, the DGM‐based sensor enables reliable and high‐resolution monitoring of diverse physiological activities, including pulse waveforms, respiratory rate, heartbeat, and swallowing. Additionally, its broad linear response range facilitates simplified circuit design and ensures stable signal acquisition, demonstrating potential for wearable health monitoring applications.

## 3 Conclusion

We developed a DGM‐based flexible piezoresistive pressure sensor that achieves both high sensitivity (69.8 kPa⁻¹) and a broad linear sensing range up to 300 kPa (*R* ^2^ ≈ 0.998). The dual‐graded compressible structure, in conjunction with an optimized MWCNTs content in the TPU matrix, forms a continuously deformable interface that promotes pressure‐dependent increases in contact area and tunneling conduction, thereby enabling stable and linear signal modulation. The sensor further exhibits rapid response‐relaxation time (≈5 ms in total), high‐frequency detection up to 200 Hz, and good mechanical durability over 5 000 loading cycles. In practical physiological monitoring, the DGM‐based sensor accurately captures radial pulse waveforms, respiration rate, heartbeat‐induced vibrations, and swallowing, demonstrating its potential for multimodal health monitoring. This work establishes a scalable and cost‐effective sensing approach with broad applicability, paving the way for future wearable diagnostic platforms and intelligent biomedical technologies.

## 4 Experimental Section

### Materials

TPU (Mw. 100,000, 1185A) obtained from Wespin Technology Co., Ltd. MWCNTs (diameter: 10–20 nm, length: 15–30 µm, Figure S1, Supporting Information) were purchased from Shenzhen Suiheng Technology Co., Ltd. N,N‐dimethylformamide (DMF, AR) was purchased from Maclin biochemical Technology Co., Ltd. Sandpaper templates were obtained from South Science Grind industrial Ltd. PET double‐sided tape (thickness: 6 µm) and PET single‐sided tape (thickness: 20 µm) were purchased from Darit Tape Co., Ltd.

### Fabrication of the DGM‐Based Piezoresistive Sensors—Fabrication of the TPU@MWCNTs Sensitive Films

First, 2 g of TPU was dissolved in 100 g of DMF by heating and stirring at 80°C for ≈2 h until a homogeneous solution was obtained. Then, 0.2 g of MWCNTs (9 wt.% of the total mass of TPU and MWCNTs) was added and ultrasonically treated using an ultrasonic homogenizer (SCIENTZ‐IID) at 120 W for 2 h to ensure uniform dispersion. The prepared TPU@MWCNTs solution was then cast onto a sandpaper mold (10 000 grits) with an area of 4.5 × 4.5 cm^2^ and dried at 80 °C for 5 h. Finally, the TPU@MWCNTs sensitive film (≈180 µm thick) was obtained by peeling it off from the sandpaper mold. TPU@MWCNTs films with different MWCNTs contents (6 wt.% and 12 wt.%) were fabricated following the same procedure, adjusting only the MWCNTs content accordingly (Figure 1a).

### Fabrication of the DGM‐Based Piezoresistive Sensors—Encapsulation of the DGM‐Based Sensors

The DGM‐based sensors consist of two TPU@MWCNTs sensitive films separated by a ring‐shaped spacer, along with two packaging layers. The TPU@MWCNTs films were patterned into a circular shapes (diameter: 6 cm) using an ultraviolet laser (355 nm, YLCF65UV). A PET double‐sided tape was cut into a hollow ring frame (outer diameter: 8 mm, inner diameter: 5.5 mm) and used as the spacer layer. Finally, the top sensitive film, the PET spacer, and the bottom sensitive film were assembled and encapsulated using a 10 µm‐thick PET single‐sided tape, forming the DGM‐based flexible piezoresistive pressure sensor.

### FEA Simulation

FEA was performed using Abaqus 2022 software. The DGM‐based TPU@MWCNTs films were modeled as incompressible neo‐Hookean materials with a Young's modulus of ≈28 MPa. All contact interactions were defined as frictionless and non‐penetrative. The initial contact area was determined under a preload of ≈20 Pa, and the total contact area was recorded as the applied pressure increased up to 450 kPa. The resulting stress distributions under different pressure levels are presented in Figure 2a.

### Characterizations and Measurements

Morphology and dimensions of MWCNTs were characterized using transmission electron microscopy (TEM, Thermo Scientific Talos F200X G2). Morphology of the sandpaper template and TPU@MWCNTs films was examined by field‐emission scanning electron microscopy (FE‐SEM, ZEISS Sigma 300) operated at 10 kV. The sheet resistance of the sensitive films was measured using a handheld four‐probe tester (M‐3, Suzhou Jingge Electronics). External pressure was precisely applied and measured using a force gauge mounted on a computer‐controlled stage (XLD‐20E, Jingkong Mechanical Testing Co., Ltd). For the high frequency dynamic test, the sensor was fixed onto a mounting component, and the high‐frequency vibration was applied through the terminal metal rod of a vibration generator (SA‐JZ002, Shiao Tech, Figure S2, Supporting Information). A static preload of ≈100 kPa was applied to ensure stable initial contact between the sensor and the vibration rod. All output currents were recorded with a digital source meter (Keithley 2400) under an applied voltage of 1 V. The wavelet transform maps were plotted using MATLAB, and the relevant code has been provided in the Supporting Information. All application tests were non‐invasive and conducted on healthy adult volunteers with informed consent, in accordance with institutional guidelines. No ethics approval was required.

## Conflict of Interest

The authors declare no conflict of interest.

## Author Contributions

N. B. and D.X. contributed equally to this work. N.B. conceived the idea, guided the experiments, analyzed the data, and wrote the manuscript. D.X. conducted the majority of the experiments. Z.S., C.G., and L.Z. contributed to experimental characterization and data acquisition. L.H., G.L., and Y.C. assisted with the application demonstrations of the sensors. X.Q. and J.Z. polished the manuscript. D.W. and W.W. supervised the overall project. All authors reviewed and approved the final version of the manuscript.

## Supporting information



Supporting Information

## Data Availability

The data that support the findings of this study are available from the corresponding author upon reasonable request.
