# Supplementary material for: Dual‐Graded Microstructure Engineering for Flexible Piezoresistive Sensors with High Sensitivity and Broad Linear Range in Physiological Monitoring
Source: Adv Sci (Weinh). 2025 Jul 14;12(38):e07135. doi: 10.1002/advs.202507135 (PMC12520465; doi:10.1002/advs.202507135)
Supplement: Supplementary file 1 — Supporting Information [file ADVS-12-e07135-s001.docx]

**Dual-Graded Microstructure Engineering for Flexible Piezoresistive Sensors with High Sensitivity and Broad Linear Range in Physiological Monitoring**

Ningning Bai^#,*,1,2^, Dandan Xu^#,1^, Zhuang Su^1^, Gangqiang Li^3^, Lilong He^3^, Yanli Chen^3^, Chengxi Guo^1^, Linxuan Zhou^1^, Xianming Qin^1,2^, Ji Zhang^1^, Daowei Wu^*,4^ and Weidong Wang^*,1,2^

^1^School of Mechano-Electronic Engineering, Xidian University, Xi’an 710071, P.R. China

^2^State Key Laboratory of Electromechanical Integrated Manufacturing of High-Performance Electronic Equipment, Xi’an 710071, China;

^3^Xi’an Chuanglian Uitrasound Technology Co., Ltd 710065, Xi’an, P.R. China

^4^Xi’an Microelectronics Technology Institute, Xi’an, 710054, P.R. China

^#^ These authors contributed equally

^*^ Corresponding authors: nnbai@xidian.edu.cn (N. B.); [wudaowei1220@163.com](mailto:wudaowei1220@163.com) (D.W.); wangwd@mail.xidian.edu.cn (W. W.)

The file includes:

Supplementary Text 1. Basic MATLAB code for WSST time–frequency analysis.

Table S1. Comparison of sensing performance among our and other piezoresistive sensors

Figure S1. Transmission electron microscopy (TEM) images of MWCNTs with an outer diameter of 10–20 nm at different magnifications.

Figure S2. Schematic of the high-frequency vibration test setup for evaluating the dynamic response of the sensor under periodic loading.

Figure S3. SEM image of the sandpaper template.

Figure S4. Cross-sectional SEM images of TPU@MWCNTs films.

Figure S5. SEM images of TPU@MWCNTs films templated by sandpaper.

Figure S6. Effect of micro-structure size on sensing performance.

Figure S7. Mechanical properties of TPU@MWCNTs sensitive films containing 6, 9, and 12 wt. % MWCNTs, respectively.

Figure S8. Dynamic mechanical stability of the DGM-based sensor under 5,000 loading–unloading cycles at a frequency of 5 Hz.

Figure S9. Arterial pulse monitoring using the DGM-based flexible piezoresistive sensor after exercise.

Figure S10. Demonstration of dynamic response monitoring using the sensor.

**Supplementary Text 1. Basic MATLAB code for WSST time–frequency analysis.**

The MATLAB script below illustrates the WSST-based time–frequency analysis of physiological signals. The input data, recorded after exercise, consists of alternating time and signal values. A high-pass filter is applied to remove low-frequency noise before performing WSST.

**The code is as follows:**

| clc;  clear;  *% Load data*  data = textread('C:\Desktop\file name.txt', '%f');  t = data(1:2:end)'; *% Time data*  y = data(2:2:end)'; *% Signal data*  *% Calculate sampling frequency*  N = length(t);  fs = N / t(end); *% Sampling frequency*  Ts = 1 / fs;  *% High-pass filter to remove low-frequency drift*  wp = 0.001 / (fs / 2); *% Passband edge*  ws = 0.1 / (fs / 2); *% Stopband edge*  alpha_p = 0.1; *% Passband ripple (dB)*  alpha_s = 25; *% Stopband attenuation (dB)*  [N4, wn] = buttord(wp, ws, alpha_p, alpha_s);  [b, a] = butter(N4, wn, 'high');  filtered_y = filter(b, a, y);  *% Remove data before t = 0, if any*  start_idx = find(t > 0, 1);  t = t(start_idx:end);  filtered_y = filtered_y(start_idx:end);  *% Apply WSST (Wavelet Synchrosqueezed Transform)*  [sst, f] = wsst(filtered_y, length(t) / t(end));  *% Plot WSST time-frequency representation*  figure;  pcolor(t, f, abs(sst));  shading flat;  xlabel('Time (s)');  ylabel('Frequency (Hz)');  title('WSST Time-Frequency Representation');  colorbar; |
| --- |

**Table S1. Simulated contact area variations of different microstructured configurations under low and high applied pressures.**

| **Configuration** | **Pressure (kPa)** | ***A*_c_/*A*_0_** | $\frac{\left( \text{A}\text{c}\text{/}\text{A}\text{0} \right)\text{450}}{\left( \text{A}\text{c}\text{/}\text{A}\text{0} \right)\text{0.15}}$ |
| --- | --- | --- | --- |
| Flat-to-graded | 0.15 | 0.4 | 21 |
|  | 450 | 8.4 |  |
| Larger graded-to-graded | 0.15 | 0.45 | 53 |
|  | 450 | 24 |  |
| Finer graded-to-graded | 0.15 | 0.2 | 165 |
|  | 450 | 33 |  |

**Table S2. Comparison of sensing performance among our and other** **piezoresistive sensors.**

| **No.** | **Microstructure** | **Sensitivity (kPa^–1^)** | **Linearity range (kPa)** | **Linear sensing factor *S*_p_**  **(*S*_p_= *S*·Δ*P*)** | **Response/**  **relaxation time (ms)** | **Ref.** |
| --- | --- | --- | --- | --- | --- | --- |
| 1 | Microdome | 53 | 0.06~ 0.96 | 48 | 38/19 | 9 |
| 2 | Double-sided pyramids | 24.6 | ＜1400 | 34440 | 8.4/9.2 | 33 |
| 3 | Interlocked porous domes | 3788.3 | ＜6 | 2730 | 100 /- | 35 |
| 4 | Janus conductive structure | 4.1 | ＜1000 | 4110 | 100/50 | 36 |
| 5 | Micropillar and fabric structure | 6.4 | ＜800 | 5120 | 4/- | 37 |
| 6 | Micropyramidal array | 26.6 | 0.02-600 | 15960 | 40/20 | 38 |
| 7 | Micropyramids | 0.04 | 10 ~ 500 | 20 | 20 /- | 39 |
| 8 | Hierarchical microstructure | 134 | ＜1.5 | 201 | 20 /- | 40 |
| 9 | Hierarchical  structures | 21.7 | 0.003 ~ 43 | 932 | NA | 41 |
| 10 | Double ‘zig-zag’ and dotted surface | 4.3 | 0.6 ~ 60.4 | 257 | 214 /- | 42 |
| 11 | Random cracks | 12.3 | ＜200 | 2460 | NA | 43 |
| 12 | Gaussian-curve-shaped microstructure | 1.8 | 0.02 ~ 30 | 53 | 25/50 | 44 |
| **13** | **Dual-graded microstructure** | **69.8** | ＜**300** | **20940** | **1/4** | **Our work** |


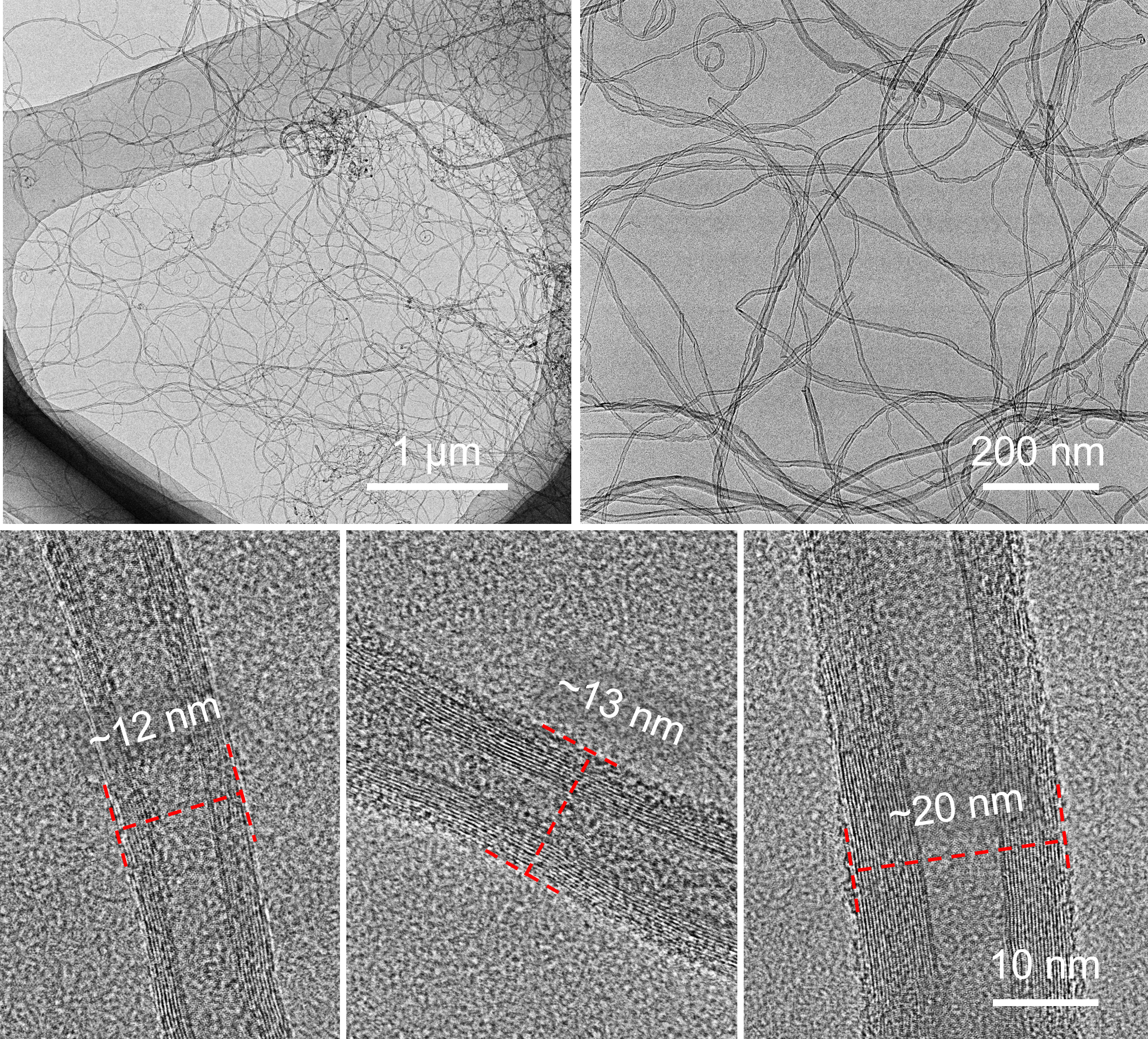


**Figure S1**. **Transmission electron microscopy (TEM) images of MWCNTs with an outer diameter of 10–20 nm at different magnifications.**


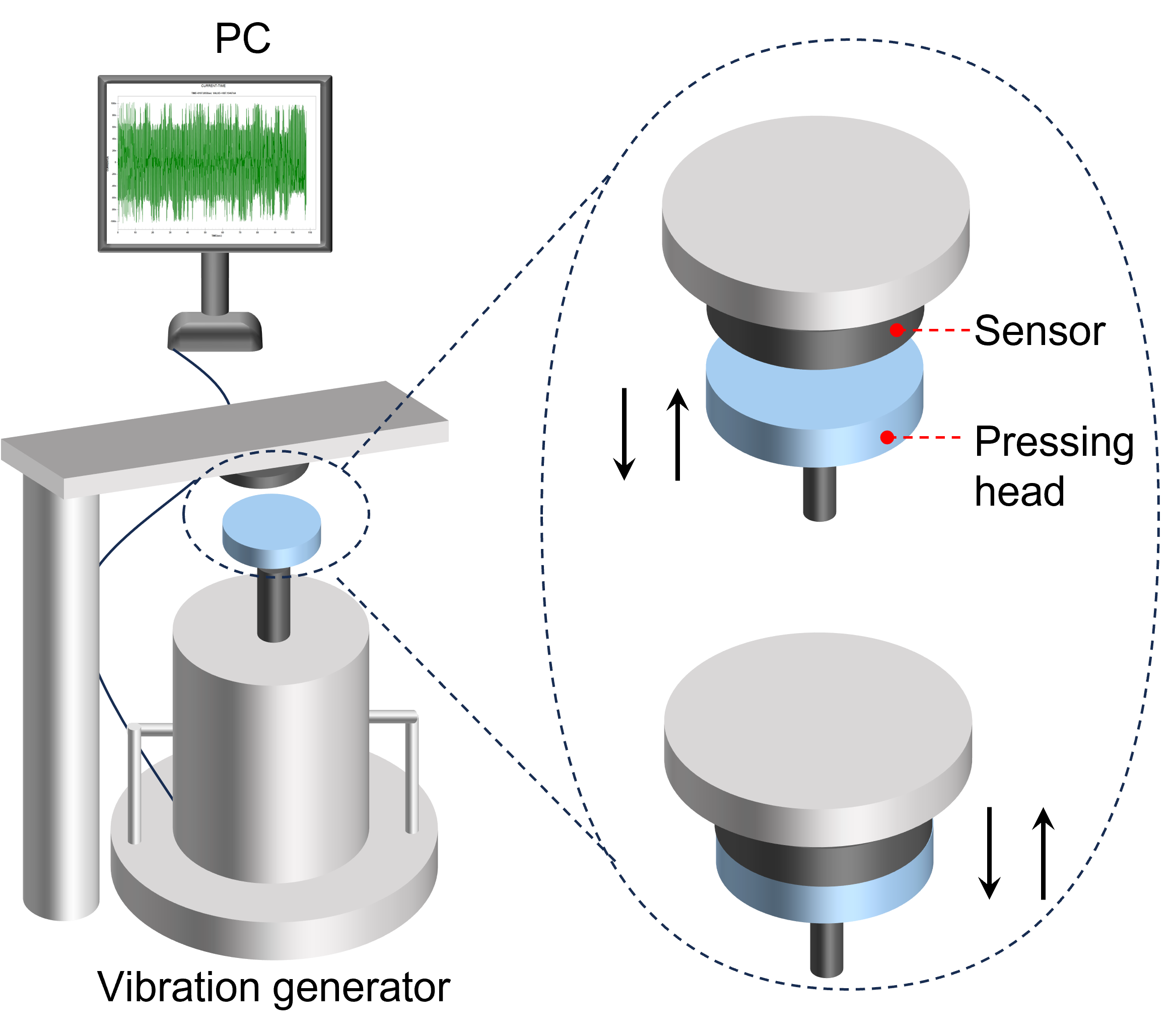


**Figure S2**. **Schematic of the high-frequency vibration test setup for evaluating the dynamic response of the sensor under periodic loading.**


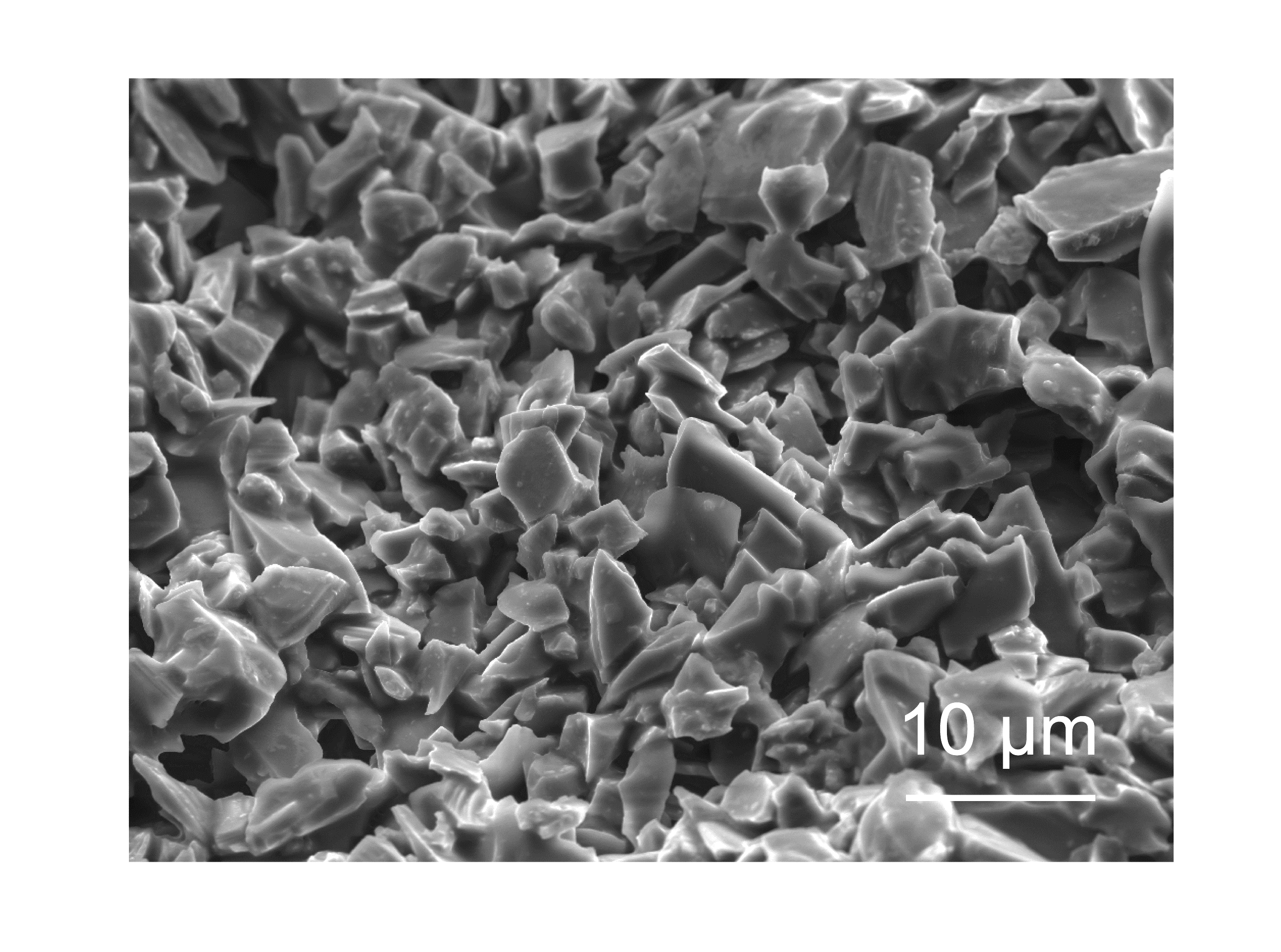


**Figure S3**. **SEM image of the sandpaper template.**


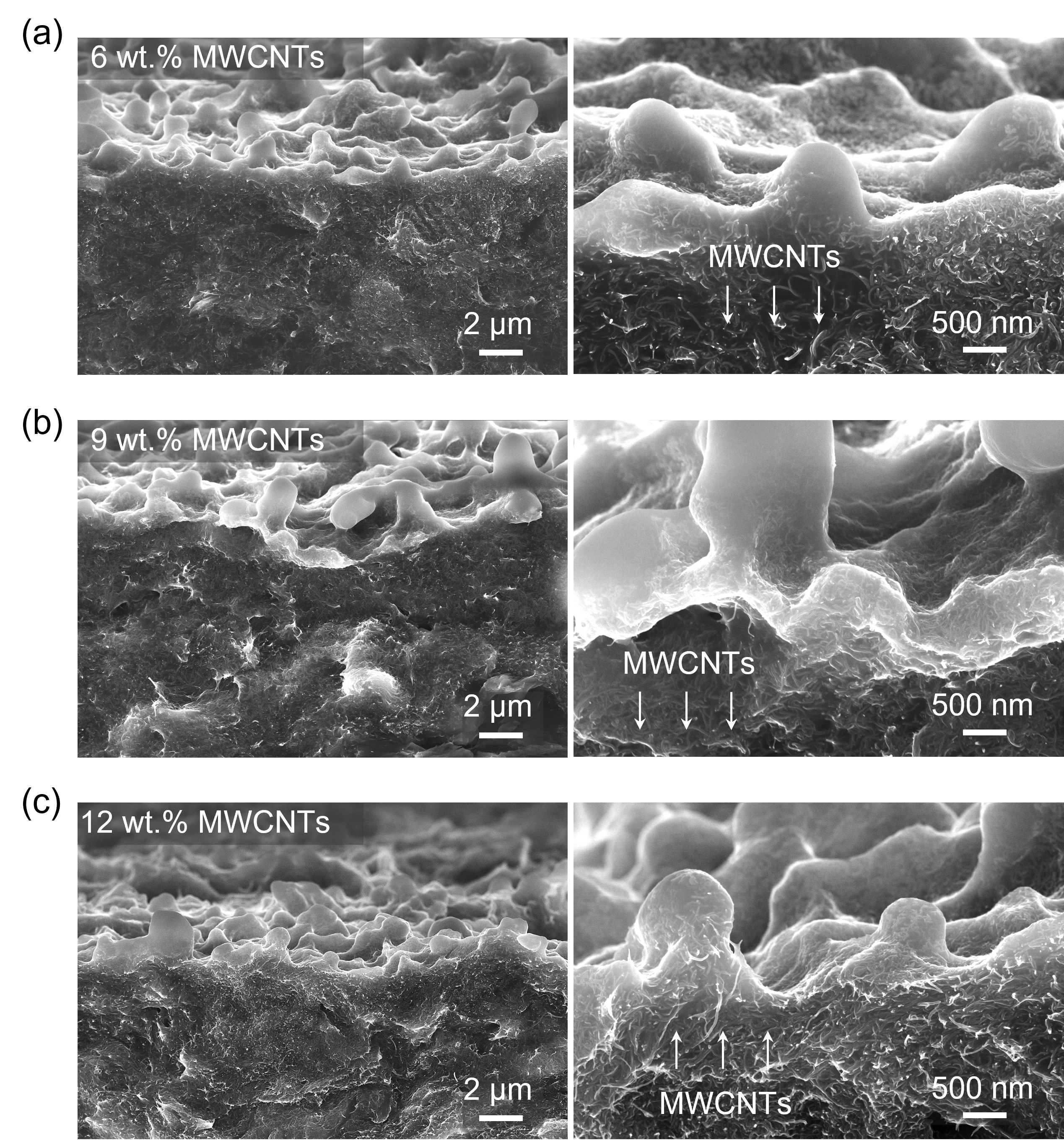


**Figure S4**. **Cross-sectional SEM images of TPU@MWCNTs films containing** (a) 6 wt.% MWCNTs, (b) 9 wt.% MWCNTs, and (c) 12 wt.% MWCNTs at different magnifications.


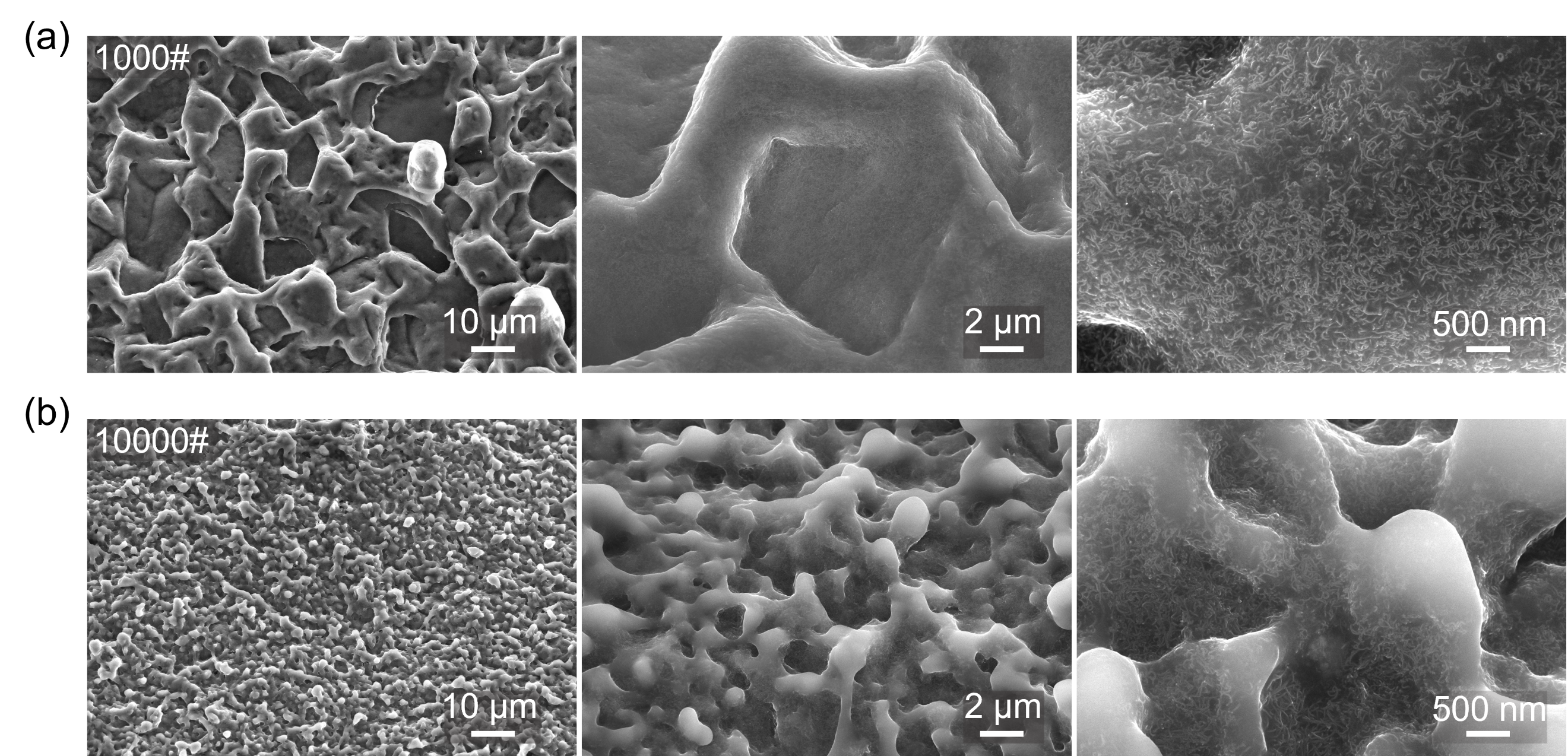


**Figure S5.** **SEM images of microstructured TPU@MWCNTs films fabricated using sandpapers of different grit numbers**: (a) 1000-grit, forming large ridges (~10–20 μm) with spacings of ~10–25 μm; (b) 10,000-grit, forming finer ridges (~500 nm–3 μm) with spacings of ~1–3 μm.


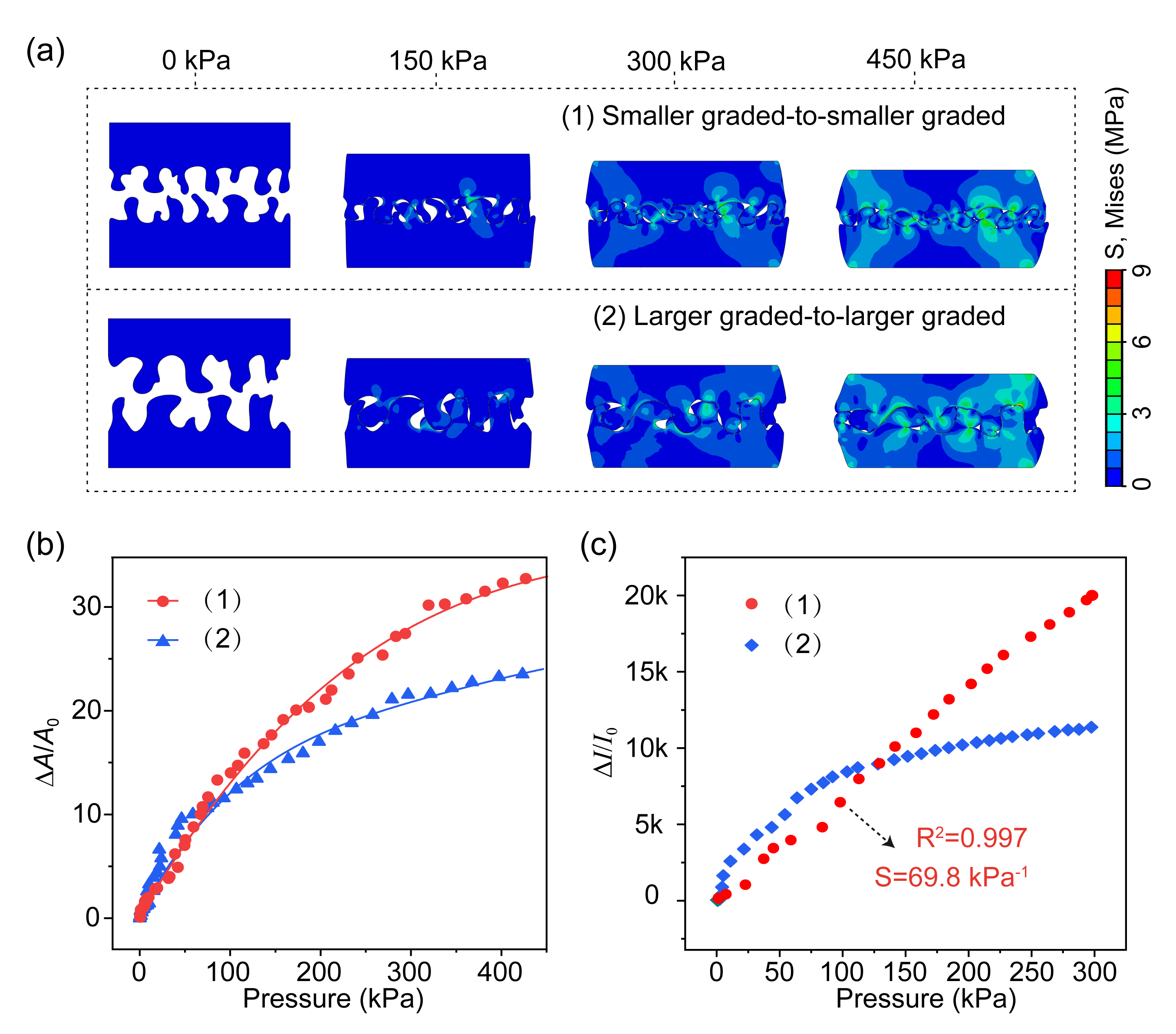


**Figure S6. Effect of micro-structure size on sensing performance.** (a) Stress distribution of simulation for smaller graded-to-graded and larger graded-to-graded configurations under pressures up to 450 kPa. (b) Normalized change in contact area for these two configurations under a broad sensing range over 450 kPa. (c) Change in current for the sensors (9 wt.% MWCNTs) with smaller graded-to-graded and larger graded-to-graded configurations under pressures up to 450 kPa.


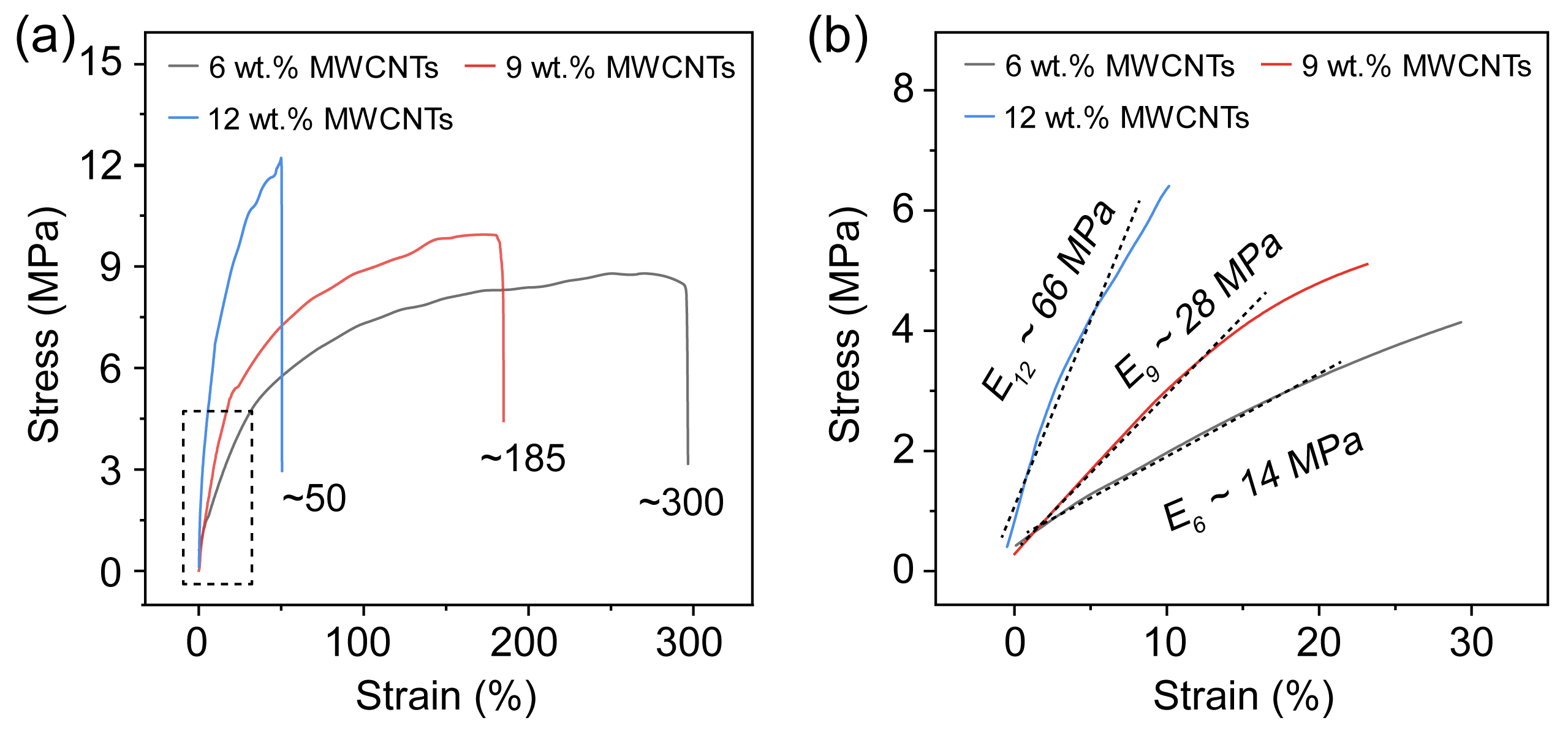


**Figure S7. Mechanical properties of TPU@MWCNTs sensitive films containing 6, 9, and 12 wt. % MWCNTs, respectively.** (a) Stress–strain curves. (b) Corresponding Young’s moduli.


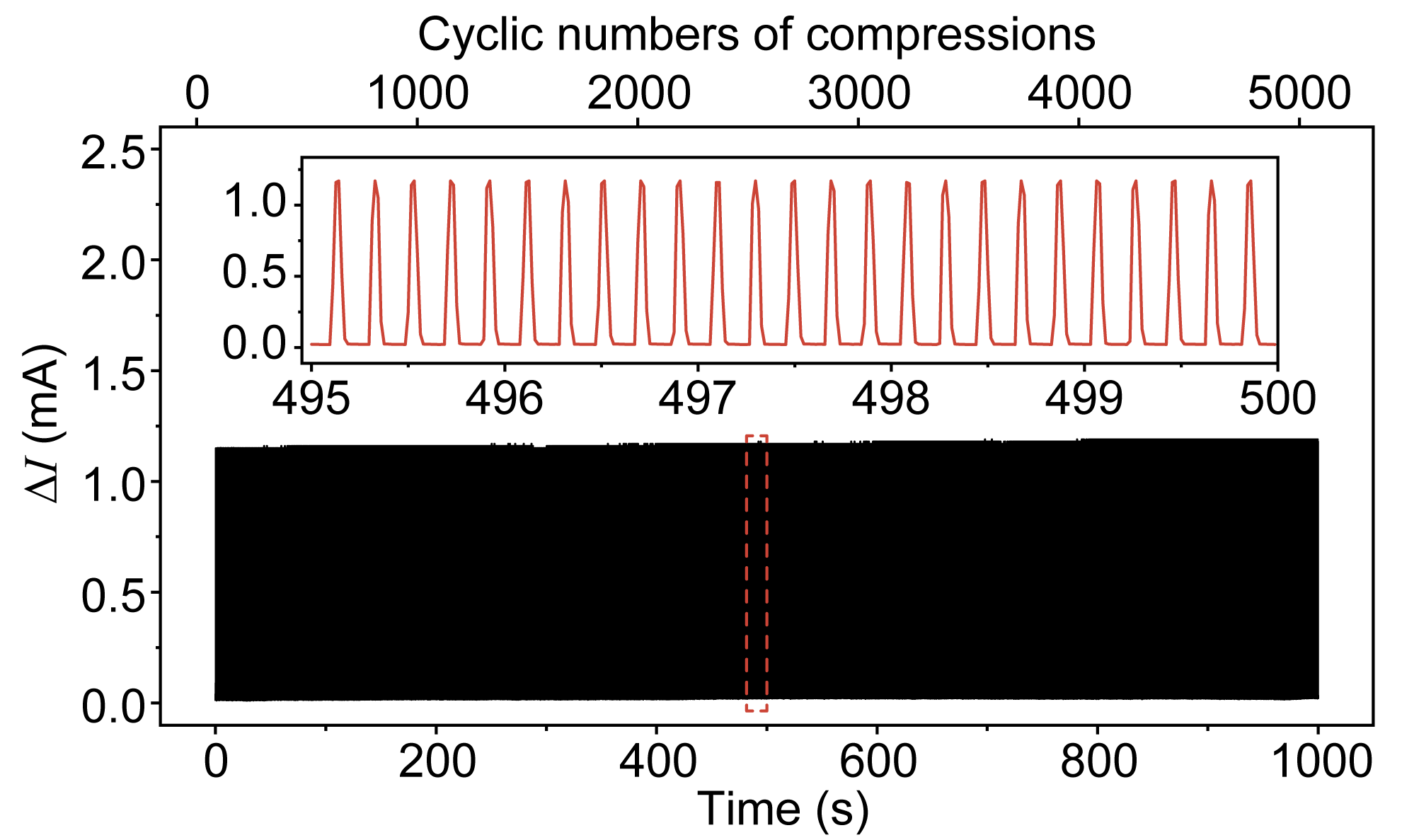


**Figure S8. Dynamic mechanical stability of the DGM-based sensor under 5,000 loading–unloading cycles at a frequency of 5 Hz.**


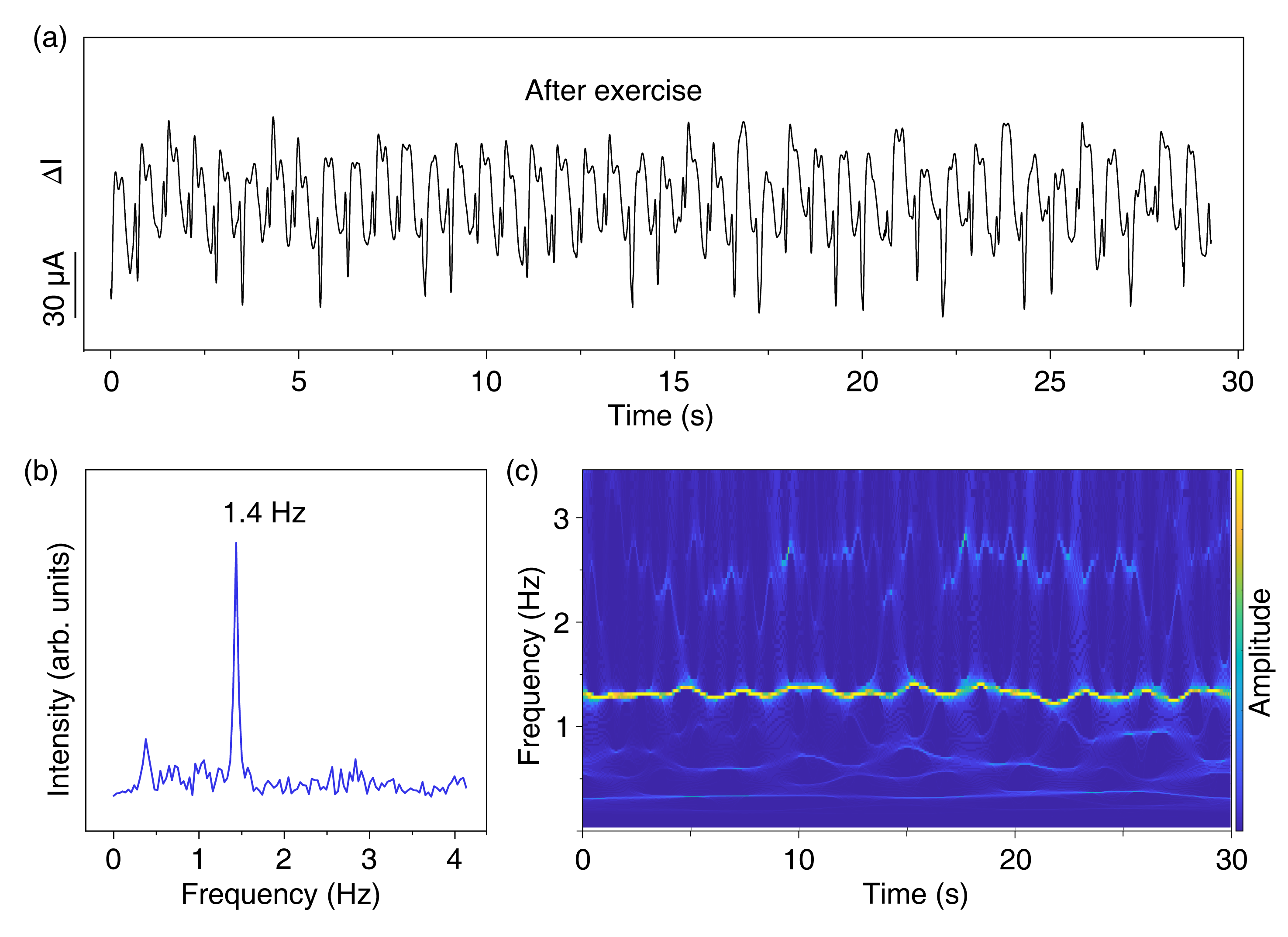


**Figure S9. Arterial pulse monitoring using the DGM-based flexible piezoresistive sensor after exercise.** (a) Time-domain radial-artery pulse signals. (b) Extracted pulse frequency. (c) Wavelet transform of the signals in panel (a), illustrating temporal evolution of frequency components.


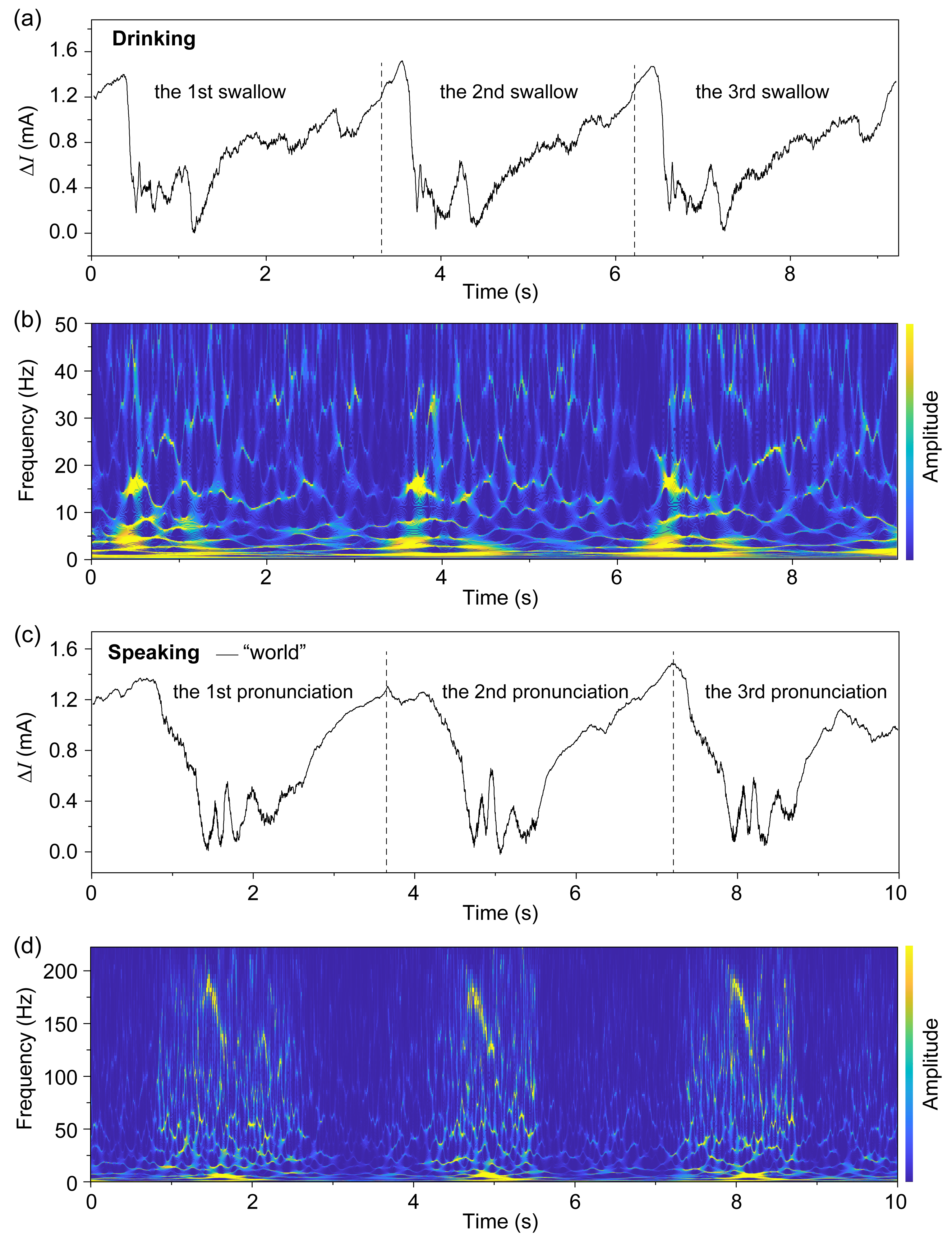


**Figure S10. Demonstration of dynamic response monitoring using the sensor**. (a) Real-time current signals during three successive swallows. (b) Corresponding wavelet time-frequency analysis of panel (a). (c) Real-time current signals while pronouncing the word “world”. (d) Corresponding time–frequency analysis of panel (c).
